# Surveillance of health care workers for multidrug-resistant organisms in faecal samples in a tertiary care cancer centre

**DOI:** 10.1186/1753-6561-5-S6-P239

**Published:** 2011-06-29

**Authors:** SK Biswas, R Kelkar, S Tandon

**Affiliations:** 1 microbiology, TATA Memorial Centre, Mumbai, Mumbai, India; 2 Pulmonary medicine, TATA Memorial Centre, Mumbai, Mumbai, India

## Introduction / objectives

Antimicrobial resistance among nosocomial pathogens is a significant problem in most clinical settings adding to the cost of medical care and the morbidity and mortality of patients. *Enterobacteriaceae* carrying extended spectrum β-lactamases(ESBLs), Vancomycin resistant *Enterococcus* (VRE), methicillin resistant *Staphylococcus aureus* (MRSA) have emerged as significant pathogens. Many of the health care workers, who come in contact with patients regularly, are carriers of many of these organisms. They are potential source of transmission of these organisms.

This study was undertaken to determine the extent of prevalence of faecal carriers of multi-drug resistant organisms among health care workers.

## Methods

A total of 211 faecal samples were processed on MacConkey agar with Ceftazidime, bile esculin azide agar, Salmonella – Shigella agar and blood agar as per standard microbiological methods. Identification of the organisms and ESBL production was done as per CLSI guidelines.

## Results

Of the 211 faecal samples received, 66(31.2 %) had growth of VRE’s , 99(46.9 %) had ESBLs and 13(6.6 %) had Salmonella spp.

## Conclusion

Although several studies have been reported on faecal carriage of ESBLs and VRE’s in patients, there has been no report on health care workers. Since we have high incidence of ESBLs and VRE’s in our hospital, this study was undertaken. Health care workers have a high risk of transmitting as many of our patients are neutropenic. Many of our patients are VRE and ESBLs carriers who have got the infection from health care workers. This needs to be further investigated.

## Disclosure of interest

None declared.

